# Lattice Matching and Microstructure of the Aromatic Amide Fatty Acid Salts Nucleating Agent on the Crystallization Behavior of Recycled Polyethylene Terephthalate

**DOI:** 10.3390/molecules29133100

**Published:** 2024-06-28

**Authors:** Tianjiao Zhao, Fuhua Lin, Yapeng Dong, Meizhen Wang, Dingyi Ning, Xinyu Hao, Jialiang Hao, Yanli Zhang, Dan Zhou, Yuying Zhao, Jun Luo, Jingqiong Lu, Bo Wang

**Affiliations:** 1 School of Chemical Engineering and Technology, Taiyuan University of Science and Technology, Taiyuan 030024, China; s202221211044@stu.tyust.edu.cn (T.Z.); s202221211035@stu.tyust.edu.cn (Y.D.); s202321111073@stu.tyust.edu.cn (M.W.); 202021070217@stu.tyust.edu.cn (D.N.); 201921050134@stu.tyust.edu.cn (X.H.); s202221211046@stu.tyust.edu.cn (J.H.); 2021051@tyust.edu.cn (Y.Z.); 2021053@tyust.edu.cn (D.Z.); 1991030@tyust.edu.cn (Y.Z.); 2015020@tyust.edu.cn (J.L.); 2 School of Traffic Engineering, Shanxi Vocational University of Engineering Science and Technology, Jinzhong 030619, China; linfuhua@sxgkd.edu.cn; 3 Guangzhou Fibre Product Testing and Research Institute, Guangzhou 510220, China; luoj@iccas.ac.cn

**Keywords:** recycled polyethylene terephthalate, lattice matching, microstructure, crystallization behavior, mechanical properties, thermal properties

## Abstract

To solve the decrease in the crystallization, mechanical and thermal properties of recycled polyethylene terephthalate (rPET) during mechanical recycling, the aromatic amide fatty acid salt nucleating agents Na-4-ClBeAmBe, Na-4-ClBeAmGl and Na-4-ClAcAmBe were synthesized and the rPET/nucleating agent blend was prepared by melting blending. The molecular structure, the thermal stability, the microstructure and the crystal structure of the nucleating agent were characterized in detail. The differential scanning calorimetry (DSC) result indicated that the addition of the nucleating agent improved the crystallization temperature and accelerated the crystallization rate of the rPET. The nucleation efficiencies (*NE*) of the Na-4-ClBeAmBe, Na-4-ClBeAmGl and Na-4-ClAcAmBe were increased by 87.2%, 87.3% and 41.7% compared with rPET which indicated that Na-4-ClBeAmBe and Na-4-ClBeAmGl, with their long-strip microstructures, were more conducive to promoting the nucleation of rPET. The equilibrium melting points ($T_{m}^{0}$) of rPET/Na-4-ClBeAmBe, rPET/Na-4-ClBeAmGl and rPET/Na-4-ClAcAmBe were increased by 11.7 °C, 18.6 °C and 1.9 °C compared with rPET, which illustrated that the lower mismatch rate between rPET and Na-4-ClBeAmGl (0.8% in b-axis) caused Na-4-ClBeAmGl to be the most capable in inducing the epitaxial crystallization and orient growth along the b-axis direction of the rPET. The small angle X-ray diffraction (SAXS) result proved this conclusion. Meanwhile, the addition of Na-4-ClBeAmGl caused the clearest increase in the rPET of its flexural strength and heat-distortion temperature (*HDT*) at 20.4% and 46.7%.

## 1. Introduction

Polyethylene terephthalate (PET) has the advantages of transparency, chemical resistance and thermal stability, and has been widely used in packaging films, textile fibers and beverage bottles [1,2]. But the extensive use of the PET in daily life has caused serious environmental pollution [3,4]. Therefore, recycled polyethylene terephthalate (rPET) has received increasing attention [5]. At present, chemical recycling and mechanical recycling have been applied in the field of PET recycling. Mechanical recycling involves secondary processing and reuse through cleaning, crushing, and blending with necessary additives and extruder granula, which is considered the most economical and convenient recycling method in the field of PET recycling [6,7].

**Figure 1:**
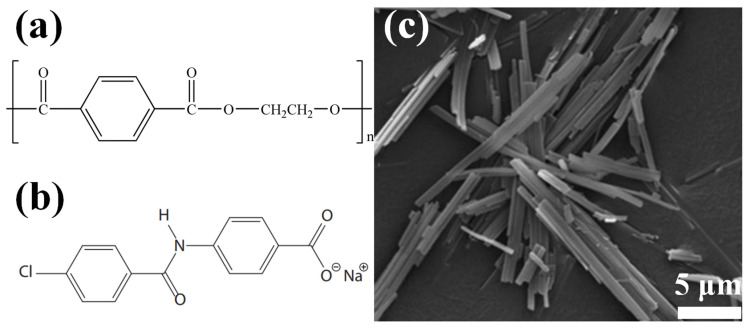
(**a**) The molecular structure of the rPET; (**b**) the molecular structure of Na-4-ClBeAmBe; (**c**) the microstructure of Na-4-ClBeAmBe [26].

However, the molecular chains of rPET are easy to break at high temperatures during mechanical recycling due to the presence of ester bonds in the molecular chains of rPET (Figure 1a) which leads to a reduction in the order degree of the rPET molecular chains [8]. Because of this, the crystallization properties of rPET decrease sharply which cause the mechanical and thermal properties of the rPET to greatly decrease [9]. As a commonly used method for polymer modification, the blending modification has the advantages of simplicity and cost-effectivenes, and has been widely applied in the crystallization behavior modifications of rPET [10]. The nucleating agent has the advantages of having a small addition amount and excellent effects, and was therefore considered to be an effective additive for blending modification within rPET [11]. The nucleating agent can transform the nucleation mode of rPET from homogeneous nucleation to heterogeneous nucleation. Meanwhile, the nucleating agent also provides the external surface for rPET to reduce the nucleation free energy of rPET [12,13]. This effect greatly reduces the energy required for rPET homogeneous nucleation, which can affect the order degree and increase the crystallization rate and the crystal density.

It is well known that the rPET crystallization process consists of nucleation and crystal growth [14]. Among numerous methods of crystal growth, epitaxial crystallization has the special function of inducing the directional arrangement of molecular chains which causes an increase in the degree of order of the rPET molecular chains, thereby improving the crystallization properties of rPET and ultimately obtaining a rPET with superior mechanical and thermal properties [15,16]. Epitaxial crystallization can be induced and the crystals of the rPET can grow along a specific direction when the structure of the nucleating agent has a lattice-matching relationship with the rPET, but the mismatch rate between the nucleating agent and the rPET must be less than 15% [17]. In addition, research has shown that the nucleating agent with a microstructure of a long stripe or fibre can also induce epitaxial crystallization in the polymer [18,19].

At present, research into a nucleating agent that induces epitaxial crystallization in polymers is primarily focused on inorganic materials [20]. Zhang et al. [21] discussed the effect of palygorskite (PGS) on the crystal transformation of the isotactic polybutene (iPB). The results showed that PGS can greatly accelerate the crystalline transformation of the iPB because of the lattice-matching effect of the crystal form I and the PGS. Ouchiar et al. [22] found that talc powder with a mismatch rate of less than 15% with polylactic acid (PLA) can orient the growth of PLA along the direction in the (001) plane of the talc powder, thus effectively improving the crystallization properties of PLA. Wang et al. [19] utilized bacterial cellulose (BC) with a long-strip microstructure to induce epitaxial crystallization in isotactic polypropylene (iPP). These findings demonstrate that iPP exhibits a preferential crystallization along the surface of BC at high density, thereby inhibiting the lateral growth of iPP spherulites. Furthermore, the orientation of iPP crystals was observed to occur in a direction perpendicular to the surface of the BC. As a result, the crystallization and mechanical properties of iPP were significantly improved.

But the inorganic nucleating agent has limited effectiveness in improving the crystallization behavior of rPET because of the poor compatibility in the rPET matrix [23]. On the contrary, organic nucleating agents generally have superior modification effects by reason of their good compatibility with rPET, offering them more potential application in the modification of rPET, and even more so, bringing other unexpected excellent effects [24]. Therefore, organic nucleating agents that have a lattice-matching relationship with rPET and have excellent aspect ratio microstructures may induce the production of epitaxial crystallization in the crystallization process of rPET and promote the crystal orientation of rPET, which can greatly improve the mechanical and thermal properties of rPET [25].

Milliken & Company has developed a kind of organic nucleating agent HPN 210 M. for the modification of high density polyethylene (HDPE). The principal component of the HPN 210 M. containing aromatic amide fatty acid salt is sodium 4-[(4-chlorobenzoyl) amino] benzoate (Na-4-ClBeAmBe) which has a long-strip microstructure with excellent aspect ratio (Figure 1). The research has shown that Na-4-ClBeAmBe can induce the machine-direction lamellar growth of HDPE. The addition of Na-4-ClBeAmBe can accelerate the crystallization rate of HDPE by 5.2% and remarkably improve the impact strength of HDPE [26]. Consequently, Na-4-ClBeAmBe may induce epitaxial crystallization during the crystallization process of rPET, thereby improving the crystallization properties and mechanical properties of said rPET.

However, the effect of Na-4-ClBeAmBe on the crystallization behaviour of rPET remains unclear. Therefore, exploring the influence of Na-4-ClBeAmBe on the crystallization behavior of rPET and changing the molecular structure of Na-4-ClBeAmBe to obtain the new excellent aromatic amide fatty acid salt nucleating agent based on the molecular design strategy can clarify the role of the lattice-matching relationship between the nucleating agent and rPET, the orientation growth mode of rPET crystals induced by the nucleating agent, and the effect of the microstructure of the nucleating agent on the crystallization behavior of rPET, which is greatly helpful in obtaining a new structure for an efficient aromatic amide fatty acid salt nucleating agent for rPET [27]. 

In this study, Na-4-ClBeAmBe was synthesized through chemical reaction and two types of aromatic amide fatty acid salt nucleating agent with different molecular structures were obtained by changing the functional groups of Na-4-ClBeAmBe. The three kinds of aromatic amide fatty acid salt nucleating agents were separately submitted to melting blending with rPET to prepare the rPET/aromatic amide fatty acid salt nucleating agent blend. The molecular structure, the thermal stability, the microstructure and the crystal structure of the nucleating agent was characterized in detail. The crystallization behavior of the rPET/aromatic amide fatty acid salt nucleating agent blend was characterized by differential scanning calorimetry (DSC) and small angle X-ray diffraction (SAXS). In parallel, the impact of the lattice matching, orientation growth mode and microstructure of the nucleating agent on the crystallization process of the rPET was subjected to in-depth investigation. Moreover, the mechanical and thermal properties of the blend were also discussed.

## 2. Results

### 2.1. The Characterization Analysis of the Aromatic Amide Fatty Acid Salt Nucleating Agent

**Figure 2:**
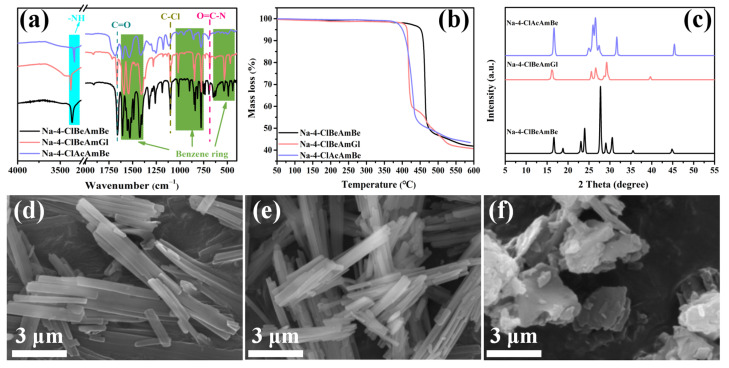
(**a**) The FTIR spectra of the sample; (**b**) The TG curve of the sample; (**c**) The XRD curve of the sample; (**d**) The microstructure of Na-4-ClBeAmBe; (**e**) The microstructure of Na-4-ClBeAmGl; (**f**) The microstructure of Na-4-ClAcAmBe.

The FTIR spectra of the sample are shown in Figure 2a. The vibrational peaks of –NH, C=O and O=C-N appear at 3460–3361 cm^−1^, 1660 cm^−1^ and 680 cm^−1^, respectively, which proves the occurrence of the acylation reaction [28]. The vibration peaks of the benzene ring skeleton present at 1606, 1529 and 1423 cm^−1^ [29]. The peaks at 900–700 cm^−1^ represent the out-of-plane bending vibration of the C-H in the benzene ring and those at 777 cm^−1^ represent the para-substitution in the benzene ring. Meanwhile, the peaks below 680 cm^−1^ are the in-plane and out-of-plane ring deformation vibration of the benzene ring. In addition, the vibration peak of C-Cl appeared at 1101 cm^−1^. The results prove that the aromatic amide fatty acid salt nucleating agents were synthesized successfully.

The TG curve of the sample is shown in Figure 2b. It can be seen that all the aromatic amide fatty acid salt nucleating agents had good thermal stability, and thermal decomposition did not occur in the range of 100–300 °C, which indicated that the chemical structure of the nucleating agent remained intact in the processing temperature of the rPET (280 °C) and can be used as the dispersing nucleating agent of rPET.

The microstructure of the sample is shown in Figure 2d–f. It can be seen that the microstructures of Na-4-ClBeAmBe and Na-4-ClBeAmGl are long-strip microstructures with excellent aspect ratios and that Na-4-ClAcAmBe is shown to have a plate microstructure. Therefore, Na-4-ClBeAmBe and Na-4-ClBeAmGl have more potential to induce epitaxial crystallization in rPET, thereby promoting the oriented growth of rPET in the crystallization process.

The XRD curve of the sample is shown in Figure 2c. It can be seen that Na-4-ClBeAmBe, Na-4-ClBeAmGl and Na-4-ClAcAmBe have different crystal structures. The sample underwent phase retrieval and quantitative analysis through Jade and then the lattice spacing of the sample was calculated through the Bragg formula (Equation (1)) [30]. The lattice parameters of the sample were obtained and are recorded in Table 1.

(1) 2dsinθ=nλ

where *d* is the interplanar spacing, *θ* is the angle between the incident X-ray and the corresponding crystal plane, *λ* is the wavelength of the X-ray and *n* is the diffraction order.

The mismatch rate between the rPET and the aromatic amide fatty acid salt nucleating agents was calculated according to Equation (2).

(2) $$\Delta =\frac{\left(d-d_{0}\right)}{d_{0}}\times 100\%$$

where *d* and *d*_0_ represent the parameters of the rPET and the aromatic amide fatty acid salt nucleating agent in the lattice matching.

The mismatch rates between the rPET and Na-4-ClBeAmBe on the b-axis and c-axis were 14.7% and 7.4%, and the rPET with Na-4-ClBeAmGl and Na-4-ClAcAmBe on the b-axis were 0.8% and 8.9%, respectively. The data indicated that Na-4-ClBeAmBe, Na-4-ClBeAmGl and Na-4-ClAcAmBe can achieve the lattice-matching condition for inducing epitaxial crystallization in rPET and that they have the potential to promote the oriented growth of rPET in the crystallization process.

### 2.2. The Crystallization Behavior of the rPET/Nucleating Agent Blend

**Figure 3:**
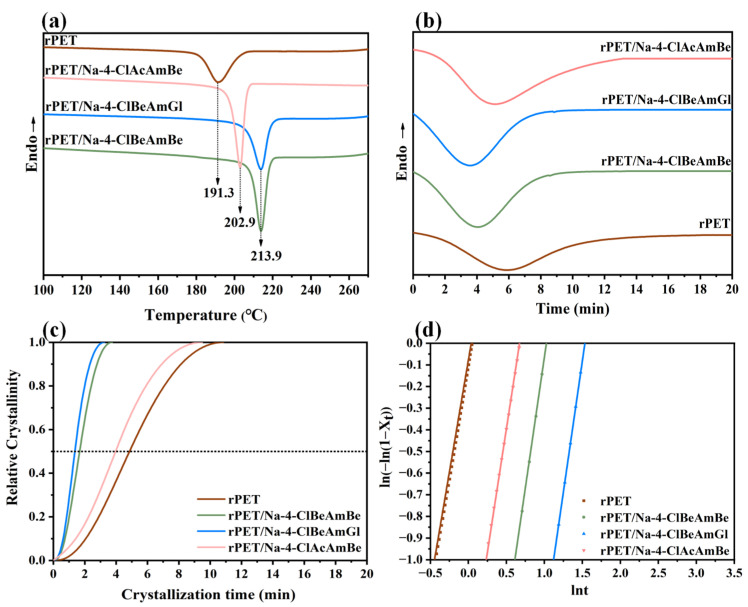
(**a**) The non-isothermal crystallization curve of the sample; (**b**) The isothermal crystallization curve of the sample; (**c**) The curve of the *X_t_* versus crystallization time of the sample; (**d**) The isothermal crystallization kinetics curve of the sample.

The non-isothermal and isothermal crystallization behaviors of the rPET/nucleating agent blend are shown in Figure 3. The non-isothermal crystallization curve of the sample is shown in Figure 3a and the degree of crystallinity (*X*) of the sample was calculated by Equation (3) and is recorded in Table 2.

(3) $$X=100\%\times \frac{\Delta H_{m}}{{\Delta H}_{m}^{0}}$$

where $\Delta H_{m}$ is the actual measured melting enthalpy and ${\Delta H}_{m}^{0}$ is the standard melting enthalpy for the complete crystallization of the rPET, which is 140.1 J/g [32].

It can be clearly seen from Figure 3a that the crystallization temperature (*T_c_*) of the rPET/Na-4-ClBeAmBe and the rPET/Na-4-ClBeAmGl blends was significantly elevated to 22.6 °C compared with the rPET, while the rPET/Na-4-ClAcAmBe was only increased to 11.6 °C. This is because the nucleating agent can provide the heterogeneous nucleation sites for the rPET thereby increasing the *T_c_* of the rPET and shortening the forming cycle of the rPET. Compared with Na-4-ClBeAmBe, Na-4-ClBeAmGl had the same effect of improving the non-isothermal crystallization behavior of the rPET while the effect of Na-4-ClAcAmBe was relatively poor. This phenomenon was probably because Na-4-ClAcAmBe, with its plate microstructure, was less effective than Na-4-ClBeAmBe and Na-4-ClBeAmGl, with their long-strip microstructures, in increasing the *T_c_* of rPET. Compared with rPET, the *X* of the rPET/Na-4-ClBeAmBe, the rPET/Na-4-ClBeAmGl and the rPET/Na-4-ClAcAmBe blends were increased by 5.3%, 6.6% and 4.4%, respectively. This result indicates that the nucleating agent can improve the crystal structure of rPET. The *X* value of the rPET/Na-4-ClBeAmGl blend was the highest, which may be due to the lower mismatch rate between Na-4-ClBeAmGl and rPET.

The isothermal crystallization curve of the rPET/nucleating agent blend at 225 °C is shown in Figure 3b. To further explore the effect of the nucleating agent on the crystallization rate of rPET, the Avrami model, which is widely applied on polymer, was selected to describe the phase transition kinetics during the crystallization process of the blend [33]. The Avrami equation belongs to the first order dynamic equation and the general form is shown in Equation (4).

(4) $$X(t)=\exp(-K_{t}t^{n})$$

where X(t) is the relative crystallinity at time *t*, *K* is the crystallization rate constant and *n* is the Avrami index.

The half-time of crystallization (*t*_1/2_) can be calculated using Equation (5) [34].

(5) $$t_{1/2}={\left(\frac{\ln2}{K}\right)}^{1/n}$$

Transform the logarithmic on both sides of Equation (3) to obtain Equation (6).

(6) ln[−ln(1−X(t)]=lnK+nlnt

The curve of the X(t) versus crystallization time is shown in Figure 3c. Figure 3d was obtained by fitting Figure 3c according to Equation (6) where the slope of the straight line is *n* and the intercept is *K*. Meanwhile, the isothermal crystallization kinetic parameters are recorded in Table 2.

As shown in Figure 3c and Table 2, the *t*_1/2_ of the rPET/Na-4-ClBeAmBe blend and the rPET/Na-4-ClBeAmGl was decreased by 65.9% and 71.9%, respectively, while the rPET/Na-4-ClAcAmBe blend was only decreased by 18.8% compared with the rPET. The *K* value represented the same tendency with the *t*_1/2_. This is because the nucleating agent can reduce the time during the random movement of the molecular chain of the blend to form critical nuclei thereby increasing the nucleation rate and thus increasing the crystallization rate. The improvement of Na-4-ClBeAmBe and Na-4-ClBeAmGl was evdiently better than in Na-4-ClAcAmBe on the *t*_1/2_ and *K* of the rPET/nucleating agent blend which may also be attributed to the long-strip microstructure of the Na-4-ClBeAmBe and the Na-4-ClBeAmGl blends. The superior effect of Na-4-ClBeAmGl compared with Na-4-ClBeAmBe may because of the lower mismatch rate between the Na-4-ClBeAmGl and the rPET which was heplful to form a more stable crystal structure and accelerate the crystallization rate [3]. The *n* of the sample was between 2 and 3 which indicated that the growth mode of the rPET/nucleating agent blend was in the form of two-dimensions. Meanwhile, the addition of the nucleating agent had no effect on the crystal growth mode of the blend [35].

### 2.3. The Nucleation Process of the rPET/Nucleating Agent Blend

The nucleation efficiency (*NE*) of the sample was calculated according to Equation (7) proposed by Fillon et al. [36].

(7) $$\mathrm{NE}=\frac{t_{c,\mathrm{NA}}-t_{c1}}{t_{c2,\max}-t_{c1}}\times 100\%$$

where *t*_*c*,*NA*_ is the crystallization peak temperature of the rPET/nucleating agent blend, *t_c_*_1_ is the crystallization peak temperature of the rPET and *t_c_*_2,*max*_ is the maximum *T_s_* of the rPET. 

**Figure 4:**
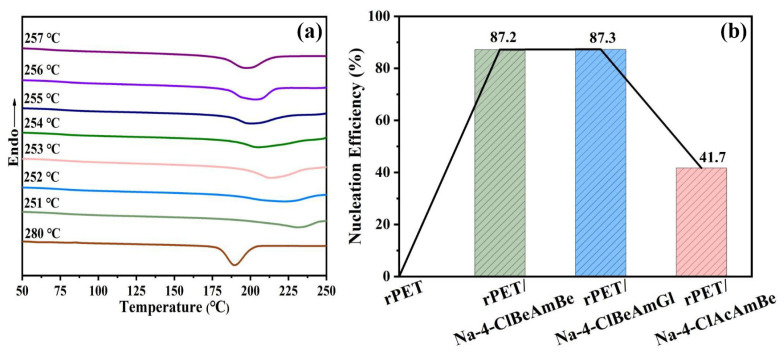
(**a**) The *SN* curve of the sample; (**b**) The *NE* of the sample.

To calculate the *NE*, the determination of the *T_s_* was required. The crystallization curve of the rPET after different heat treatments is shown in Figure 4a. The *T_c_* of the rPET began to weaken when the thermal treatment temperature was below 255 °C. When the thermal treatment temperature was in the range of 257–255 °C, the *T_c_* of the rPET rose with the decrease in the thermal treatment temperature, meaning that SN only occurred in the rPET during the cooling process. Therefore, 255 °C was selected as the *T_s_* of the rPET, and the *t_c_*_2,*max*_ was 217.2 °C [10]. 

The *NE* values are shown in Figure 4b. It can be seen that the addition of the nucleating agent significantly improved the *NE* of the rPET. This is because the nucleating agent can provide heterogeneous nucleation sites for rPET, thereby reducing the free energy of nucleation, increasing the nucleation density and thus significantly reducing the nucleation time of the rPET [37]. The improvement of the Na-4-ClBeAmBe and the Na-4-ClBeAmGl blends was remarkably, better than Na-4-ClAcAmBe, although all the nucleating agents had lattice-matching relationships with the rPET, the role of the microstructure in the nucleation stage was more pronounced, meaning the nucleating agent with the long-strip microstructure was more conducive to promoting the nucleation of the rPET. These results confirmed the conjecture of the non-isothermal crystallization behavior of the rPET/nucleating agent blend.

### 2.4. The Crystal Growth Process of the rPET/Nucleating Agent Blend

**Figure 5:**
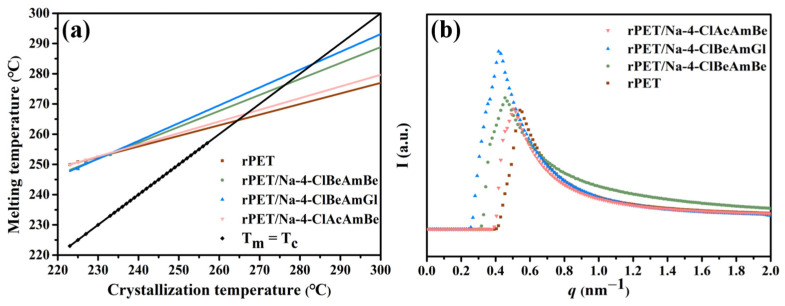
(**a**) The relationship of *T_m_* and *T_c_* for the sample; (**b**) The SAXS intensity curve of the sample.

The equilibrium melting point ($T_{m}^{0}$) of the sample was calculated according to Equation (8) and the melting point (*T_m_*) of the sample after isothermal crystallization (Table 3) [38].

(8) $$T_{m}=(1-\frac{1}{2\beta })T_{m}^{0}+\frac{1}{2\beta }T_{c}$$

where *β* is a constant related to the crystal structure and $T_{m}^{0}$ can be determined by the extrapolation of the experimental melting point *T_m_* versus *T_c_* to the line *T_m_* = *T_c_* according to Equation (8). The results were obtained and are recorded in Figure 5a and Table 3. 

Selected different isothermal crystallization temperatures (*T_c_*) and secondary melting temperatures (*T_m_*) are recorded in Table 3 and Figure 5 was then obtained based on the data from Table 3. As shown in Figure 5a and Table 3, the $T_{m}^{0}$ of rPET/Na-4-ClBeAmBe, rPET/Na-4-ClBeAmGl and rPET/Na-4-ClAcAmBe improved by 11.7 °C, 18.6 °C and 1.9 °C. Research has shown that the $T_{m}^{0}$ of the polymer is closely related to the thickness of the crystalline. A thicker crystalline can reduce the degree of freedom of the molecular chain segments, thereby strengthening the intermolecular interactions of the polymer and thus increasing the $T_{m}^{0}$ of the polymer. Therefore, the increasing thickness of a crystalline can lead to an increase in the $T_{m}^{0}$ of a polymer [39].

The result of the $T_{m}^{0}$ indicated that the addition of Na-4-ClBeAmBe and Na-4-ClBeAmGl significantly increased the crystal thickness of the rPET compared with Na-4-ClAcAmBe, which illustrated that a nucleating agent with a long-strip microstructure also had better effects during the crystal growth process. The crystal thickness of the rPET/Na-4-ClBeAmGl blend was thicker than that of the rPET/Na-4-ClBeAmBe, indicating that the lower mismatch rate between the rPET and the Na-4-ClBeAmGl (0.8% in b-axis) emphasized the capability of Na-4-ClBeAmGl to induce the epitaxial crystallization and orient the growth along the b-axis direction of the rPET.

**Figure 6:**
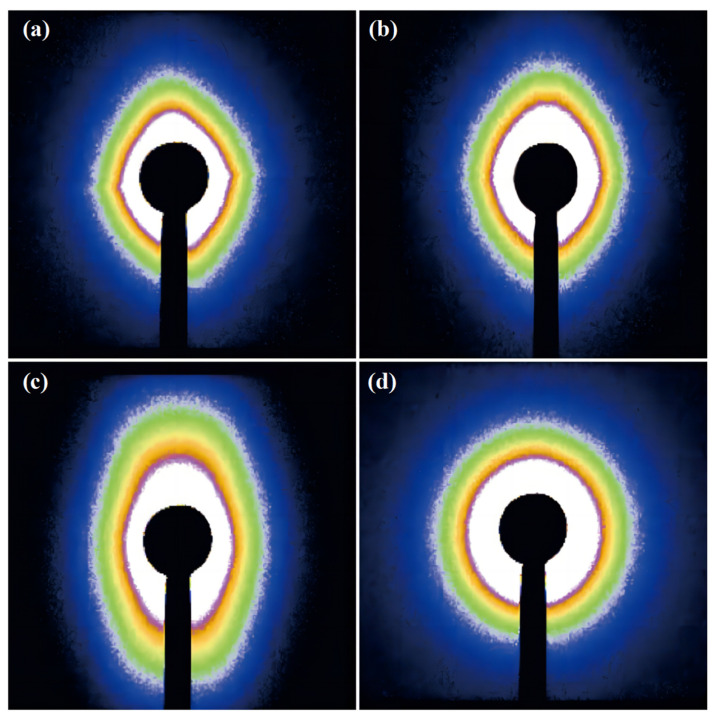
The SAXS of the samples: (**a**) rPET; (**b**) rPET/Na-4-ClBeAmBe blend; (**c**) rPET/Na-4-ClBeAmGl; (**d**) rPET/Na-4-ClAcAmBe.

The SAXS was conducted on the sample in order to further investigate the effect of the nucleating agent on the crystal orientation of rPET during the crystal growth process of the rPET and obtain Figure 6. It can be seen from Figure 6 that the rPET exhibited oriented crystallization along the tensile direction during the processing. The rPET/Na-4-ClAcAmBe blend demonstrated a concentric scattering pattern with a uniform distribution of scattering lines, indicating that Na-4-ClAcAmBe did not induce the oriented growth of the rPET. This result illustrated that although Na-4-ClAcAmBe has a lattice-matching relationship with rPET, the plate microstructure prevents the Na-4-ClAcAmBe to induce the oriented crystal growth of the rPET. The orientation of the rPET/Na-4-ClBeAmGl blend was more pronounced than the rPET/Na-4-ClBeAmBe blend. This result indicated that a lower mismatch rate is more conducive to inducing oriented crystal growth in rPET under the premise of having the same microstructure of long strips with an excellent aspect ratio. The study of the SAXS was consistent with the results of the $T_{m}^{0}$ and verifies the conjecture of the crystallization behavior of the rPET/nucleating agent blend. 

The Fit2D was used to process Figure 6 and obtain the SAXS intensity curve shown in Figure 5b. The single scattering peak of each sample can be observed from Figure 5b indicating the presence of an ordered crystal structure in the sample. The lower the angle represents the longer the period, so the lengths of the periods of the samples can be obtained from Figure 6b: rPET/Na-4-ClBeAmGl > rPET/Na-4-ClBeAmBe > rPET/Na-4 ClAcAmBe > rPET. Due to the positive correlation between the long period and the crystal thickness of the sample, the long period result further confirmed the conclusion of $T_{m}^{0}$ [40].

### 2.5. The Mechanical and Thermal Properties of the rPET/Nucleating Agent Blend

The mechanical properties of the sample are shown in Table 4. It can be seen that the flexural strengths of rPET/Na-4-ClBeAmBe, rPET/Na-4-ClBeAmGl and rPET/Na-4-ClAcAmBe were separately increased by 17.2%, 20.4% and 3.7% compared with rPET, while the tensile strengths of rPET/Na-4-ClBeAmBe, rPET/Na-4-ClBeAmGl and rPET/Na-4-ClAcAmBe were increased by 6.6%, 12.2% and 4.6%. Meanwhile, the impact strengths of rPET/Na-4-ClBeAmBe, rPET/Na-4-ClBeAmGl and rPET/Na-4-ClAcAmBe were decreased by 4.2%, 6.9% and 0.9%. This result indicated that the addition of a nucleating agent significantly increased the rigidity and fracture resistance but slightly reduced the toughness of the rPET. This is because the addition of the nucleating agent can reduce the crystal size of the rPET and form a crystal structure with compactness and uniformity, making the molecular chain segment of the rPET more difficult to move, thus improving the flexural capacity and the ability to transfer and disperse tensile stress under external forces, whilst also affecting the ability to absorb external impact energy [41]. Meanwhile, the result also proved that Na-4-ClBeAmGl with a long-strip microstructure and low mismatch rate with rPET had more pronounced effects of inducing the epitaxial growth and the oriented crystallization of the rPET, which significantly improved the rigidity and yield strength, with a slight loss of impact toughness, in the rPET through the promotion of the ordered arrangement of the rPET’s molecular chain and improvement of the crystal structure of the rPET [42].

The *HDT*s of the sample are recorded in Table 4. It can be concluded from Table 4 that the *HDT*s of rPET/Na-4-ClBeAmBe, rPET/Na-4-ClBeAmGl and rPET/Na-4-ClAcAmBe separately increased by 43.1%, 46.7% and 27.5% compared with rPET. This is because the nucleating agent can act as a physical crosslinking point, which is similar to the macromolecules, to improve the rigidity of rPET and thereby improve the *HDT* of rPET [43]. Na-4-ClBeAmGl was shown to have the most obvious trend of increasing the *HDT* of the rPET. This is because the results of the $T_{m}^{0}$ and SAXS analysis concluded that the addition of Na-4-ClBeAmGl grew the crystal thickness of the rPET the most. Research has shown that the crystal-thickening process can transform a metastable crystal into a crystal with a stable equilibrium; therefore, the addition of Na-4-ClBeAmGl can increase the crystallinity and improve the crystal structure of rPET, thereby improving the *HDT* of rPET [42].

## 3. Materials and Methods

### 3.1. Materials

The rPET (rPET-PCR80AP) was supplied by the Ningbo Jianfeng New Material Co., Ltd. (Ningbo, China). The 4-aminobenzoic acid, the 4-chlorobenzoyl chloride and the chloroacetyl chloride were purchased from Shanghai Macklin Biochemical Co., Ltd. (Shanghai, China). The glycine and sodium hydroxide (NaOH) were purchased from the Shanghai Aladdin Biochemical Technology Co., Ltd. (Shanghai, China). The acetone and alcohol were purchased from Tianjin Tianli Chemical Reagent Co., Ltd. (Tianjin, China). 

### 3.2. Preparation of the Aromatic Amide Fatty Acid Salts Nucleating Agent

4-aminobenzoic acid (0.05 mol) was dissolved in 70 mL of the acetone and then 4-chorobenzoyl chloride (0.025 mol) was added dropwise to obtain the reaction system that ensured the dripping time was 1 h. Then, the rude product was obtained by removing the acetone after reacting for 7 h. Subsequently, the rude product was washed to neutrality by acetone and deionized water and then dried at 70 °C to a constant weight to obtain the 4-chlorophenylamido-benzoic acid (4-ClBeAmBe). 4-ClBeAmBe (0.02 mol) and NaOH (0.04 mol) were dispersed into the deionized water (100 mL) with stirring for 8 h to obtain the rude product by removing the water. Consequently, the rude product was washed to neutrality using deionized water and then dried at 110 °C to obtain the Na-4-ClBeAmBe.

**Figure 7:**
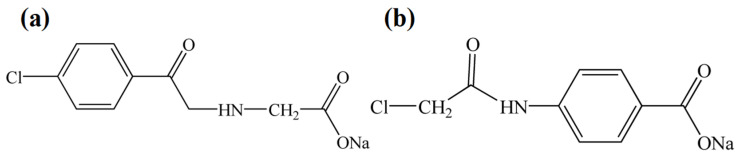
The molecular structure of the aromatic amide fatty acid salts nucleating agent: (**a**) Na-4-ClBeAmGl; (**b**) Na-4-ClAcAmBe.

Glycine (0.05 mol) was dissolved in 70 mL of acetone and then 4-chorobenzoyl chloride (0.025 mol) was added dropwise to obtain the reaction system ensuring a drip time of 1 h. Subsequently, the rude product was washed to neutrality by acetone and deionized water and then dried at 70 °C to constant weight to obtain the 4-chlorobenzoylamino-glycinate (4-ClBeAmGl). 4-ClBeAmBe (0.02 mol) and NaOH (0.04 mol) were dispersed into the deionized water (100 mL) to obtain reaction system with stirring for 8 h. The crude product was then thoroughly precipitated by adding 500 mL of alcohol to the system and dried at 60 °C to obtain sodium 4-[(4-chlorobenzoyl) amino] glycinate (Na-4-ClBeAmGl) (Figure 7a).

The 4-aminobenzoic acid (0.05 mol) was dissolved in 70 mL of acetone and then the chloroacetyl chloride (0.025 mol) was added dropwise to obtain the reaction system that ensured the dripping time was 1 h. Subsequently, the rude product was washed to neutrality by acetone and deionized water and then dried at 70 °C to constant weight to obtain 4-chloroacetylamino-benzoic (4-ClAcAmBe). The 4-ClAcAmBe (0.02 mol) and NaOH (0.04 mol) were dispersed into the alcohol (100 mL) to obtain reaction system with stirring for 8 h. Consequently, the rude product was precipitated through adding 500 mL acetone to the system and dried at 60 °C to obtain the sodium 4-[(4-chloroacetyl) amino] benzoate (Na-4-ClAcAmBe) (Figure 7b).

### 3.3. Characterization of the Aromatic Amide Fatty Acid Salt Nucleating Agent

The molecular structure of the nucleating agent was characterized using an FTIR spectrometer (Nicolet iS10, Thermo Scientific Inc., Waltham, MA, USA) using 64 scans per sample.

The thermal stability of the nucleating agent was conducted by using a thermogravimetric analyzer (TGA 1, Mettler Toledo, Zurich, Switzerland) in the range of 40 °C to 600 °C with a heating rate of 10 °C/min under a nitrogen atmosphere.

The microstructure of the nucleating agent was observed using SEM (SU5000, Hitachi Ltd., Tokyo, Japan) with an accelerating voltage of 10 kV.

The crystal structure of the nucleating agent was tested using an XRD (Rigaku Ultima IV, Rigaku Corporation, Tokyo, Japan) equipped with Cu-Kα radiation (λ = 0.154 nm) at room temperature.

### 3.4. Preparation of the rPET/Nucleating Agent Blend

The formula of the rPET/nucleating agent blend is shown in Table 5. Each component of the rPET/nucleating agent blend was mixed with a high-speed mixer (Songqing Hardware Factory, SL-500A, Yongkang, China). The rPET/nucleating agent blend was extruded and pelletized at 280 °C by a twin-screw extruder (Shanghai Xinshuo Precision Machinery Co., Ltd. WLG10A, Shanghai, China). The standard test specimens were molded by an injection machine (Shanghai Xinshuo Precision Machinery Co., Ltd. WZS10D, Shanghai, China) with an injection pressure of 15.5 MPa and 280 °C.

### 3.5. The Crystal Behavior of the rPET/Nucleating Agent Blend

The crystallization behavior of the rPET/nucleating agent blend was performed by DSC (Q1000, Waters Corporation, Milford, MA, USA) in a nitrogen atmosphere (50 mL/min). All the rPET/nucleating agent blend was eliminated the thermal history from 40 °C to 280 °C at 10 °C/min before testing.

The non-isothermal crystallization behavior: the rPET/nucleating agent blend was cooled from 280 °C to 40 °C at 10 °C/min, and then heated from 40 °C to 280 °C at 10 °C/min. 

The isothermal crystallization behavior: the rPET/nucleating agent blend was cooled from 280 °C to different isothermal temperatures (225, 227, and 230 °C) at 40 °C/min, maintained at the isothermal temperature for 50 min, then cooled to 40 °C at 10 °C/min and then heated from 40 °C to 280 °C at 10 °C/min.

**Figure 8:**
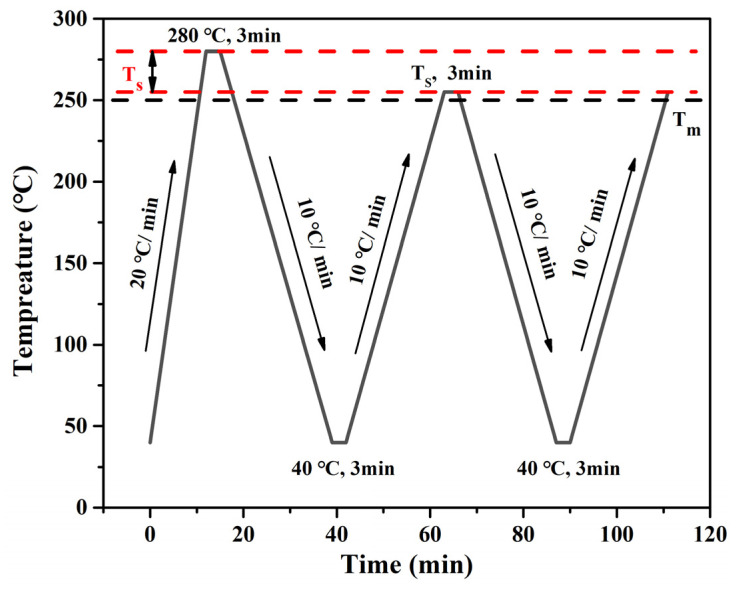
The DSC thermal procedure of the SN treatment.

The self-nucleation (SN) procedure was as follows and is shown in Figure 8: (i) Heated from 40 °C to 280 °C at 20 °C/min and held at the temperature for 3 min to eliminate the thermal history. (ii) Cooled from 280 °C to 40 °C at 10 °C/min and held for 3 min to create a “standard” thermal history. (iii) Heated from 40 °C to a pre-selected SN temperature (*T_s_*) and held for 3 min. (iv) Cooled from *T_s_* to 40 °C at 10 °C/min and held for 3 min. (v) Heated from 40 °C to melt at 10 °C/min. The aforementioned five steps were repeated at different *T_s_* [10].

### 3.6. The SAXS of the rPET/Nucleating Agent Blend

The X-ray scattering data were obtained at the beamline BL16B1 of Shanghai Synchrotron Radiation Facility (SSRF). The SAXS (Pilatus 2M, Dectris, Zurich, Switzerland) was carried out at room temperature. The radiation source was Cu Kα (λ = 0.154 nm), and the X-ray source was set at a voltage of 30 kV and a current of 30 mA. The SAXS profiles were recorded in a *2θ* range from 0.3° to 5° at a scanning rate of 0.25°/min.

### 3.7. The Mechanical Properties of the rPET/Nucleating Agent Blend

The universal testing machine (M10, Instron Company, Boston, MA, USA) was used to evaluate the tensile properties and flexural properties of the rPET/nucleating agent blend at 25 °C according to GB/T 1040-2006 and GB/T 9341-2008. The crosshead speed was set as 10 mm/min. The impact strength of rPET/nucleating agent blend was measured by an impact tester (Ceast 9050, Instron Company, Waltham, MA, USA) with a 5.5 J capacity at a maximum pendulum height (150°) at room temperature according to GB/T 1043.1-2008.

### 3.8. The Thermal Properties of the rPET/Nucleating Agent Blend 

The HDT/VICAT equipment (CEAST HV500, Instron Company, Norwood, MA, USA) was used to carry out the heat-distortion temperature (HDT) test at a heating rate of 2 °C/min and a load of 1.80 MPa according to GB/T 1634.2-2004.

## 4. Conclusions

In this work, the aromatic amide fatty acid salt nucleating agents Na-4-ClBeAmBe, Na-4-ClBeAmGl and Na-4-ClAcAmBe were synthesized through chemical reaction, which have the potential to improve the crystallization, mechanical and thermal properties of rPET. The rPET/nucleating agent blend was prepared by the melting blending method. 

The FTIR and TG confirmed the successful synthesis of the nucleating agent which can be used as the dispersing nucleating agent of the rPET, respectively. The SEM illustrated that Na-4-ClBeAmBe and Na-4-ClBeAmGl both had long-strip microstructures with excellent aspect ratios and Na-4-ClAcAmBe was shown to have a plate microstructure. The mismatch rate results can prove that the nucleating agent and rPET satisfy the lattice-matching relationship for generating epitaxial crystals.

The result of the crystallization behavior of the rPET/nucleating agent blend indicated that the nucleating agent can provide the heterogeneous nucleation sites for rPET, thereby reducing the free energy of the nucleation, increasing the nucleation density and thus improving the *T_c_* and *X* and accelerating the crystallization rate of rPET. Meanwhile, the addition of the nucleating agent had no effect on the crystal growth mode of the blend. 

Compared with rPET, the *NE*s of rPET/Na-4-ClBeAmBe, rPET/Na-4-ClBeAmGl and rPET/Na-4-ClAcAmBe were increased by 87.2%, 87.3% and 41.7%, which indicates that the role of the microstructure in the nucleation stage was more pronounced while the nucleating agents with the long-strip microstructures were more conducive to promoting the nucleation of rPET.

The $T_{m}^{0}$ of rPET/Na-4-ClBeAmBe, rPET/Na-4-ClBeAmGl and rPET/Na-4-ClAcAmBe was increased by 11.7 °C, 18.6 °C and 1.9 °C compared with rPET. This result illustrated that the nucleating agent with the long-strip microstructure also had a better effect during the crystal growth process and the lower mismatch rate between rPET and Na-4-ClBeAmGl (0.8% in b-axis) demonstrates the capability of Na-4-ClBeAmGl to induce the epitaxial crystallization and orient growth along the b-axis direction of the rPET. The SAXS result of the sample proved this conclusion.

The mechanical properties of the sample showed that the addition of the nucleating agent significantly increased the flexural strength and tensile strength but slightly reduced the impact strength of rPET. Because the improvement of the crystallization properties can lead to an increase in the mechanical properties, rPET/Na-4-ClBeAmGl had the best properties, thereby proving that Na-4-ClBeAmGl had the best effect on inducing oriented growth in rPET because of the lower mismatch rate and the long-strip microstructure with excellent aspect ratio. Meanwhile, the *HDT*s of rPET/Na-4-ClBeAmBe, rPET/Na-4-ClBeAmGl and rPET/Na-4-ClAcAmBe separately increased by 43.1%, 46.7% and 27.5% compared with rPET, which further proved this conclusion.

## Figures and Tables

**Table 1:** The crystallographic data of the sample.

Sample	Crystal System	Density (g/cm^3^)	z	Unit Cell Volume (nm^3^)	Lattice Parameters		
					a (nm)	b (nm)	c (nm)
rPET [31]	triclinic	1.455	1	0.291	0.456	0.594	1.075
Na-4-ClBeAmBe	monoclinic	0.403	1	1.228	2.215	0.518	1.161
Na-4-ClBeAmGl	monoclinic	1.158	1	0.338	0.348	0.589	1.717
Na-4-ClAcAmBe	cubic	1.413	1	0.277	0.652	0.652	0.652

**Table 2:** The non-isothermal crystallization data and isothermal crystallization kinetic parameters of the sample.

Sample	Non-Isothermal		Isothermal		
	Δ*H_m_* (J/g)	*X* (%)	*n*	*K* (min^−n^)	*t*_1/2_ (min)
rPET	29.47	21.0	2.06	2.6 × 10^−2^	4.85
rPET/Na-4-ClBeAmBe	36.91	26.3	2.41	2.1 × 10^−1^	1.65
rPET/Na-4-ClBeAmGl	38.78	27.6	2.43	3.3 × 10^−1^	1.36
rPET/Na-4-ClAcAmBe	35.57	25.4	2.28	3.0 × 10^−2^	3.94

**Table 3:** The isothermal crystallization parameters of the sample.

Sample	*T_c_* (°C)	*T_m_* (°C)	$T_{m}^{0}$ (°C)
rPET	225	250.8	264.5
	227	251.4	
	230	252.4	
rPET/Na-4-ClBeAmBe	225	248.7	276.2
	227	250.9	
	230	252.0	
rPET/Na-4-ClBeAmGl	225	248.5	283.1
	227	250.6	
	230	252.1	
rPET/Na-4-ClAcAmBe	225	250.7	266.4
	227	251.5	
	230	252.6	

**Table 4:** The mechanical and thermal properties data of the sample.

Sample	Flexural Strength (MPa)	Impact Strength (KJ/m^2^)	Tensile Strength (MPa)	HDT (°C)
rPET	66.7	3.06	54.2	69.1
rPET/Na-4-ClBeAmBe	78.2	2.93	57.8	98.9
rPET/Na-4-ClBeAmGl	80.3	2.85	60.8	101.4
rPET/Na-4-ClAcAmBe	69.7	3.03	56.7	88.1

**Table 5:** The formula of the rPET/nucleating agent blend.

Sample	rPET (wt%)	Na-4-ClBeAmBe (wt%)	Na-4-ClBeAmGl (wt%)	Na-4-ClAcAmBe (wt%)
rPET	100	0	0	0
rPET/Na-4-ClBeAmBe	100	0.3	-	-
rPET/Na-4-ClBeAmGl	100	-	0.3	-
rPET/Na-4-ClAcAmBe	100	-	-	0.3

## Data Availability

Data are contained within the article.

## References

[1] Giyahchi M., Moghimi H. (2024). Acceleration a yeast-based biodegradation process of polyethylene terephthalate microplastics by Tween 20: Efficiency, by-product analysis, and metabolic pathway Prediction. Environ. Pollut..

[2] Muszynski M., Nowicki J., Zygadlo M., Dudek G. (2023). Comparsion of catalyst effectiveness in different chemical depolymerization methods of poly(ethylene terephthalate). Molecules.

[3] Peng Y.T., Yang J., Deng C.Q., Deng J., Shen L., Fu Y. (2023). Acetolysis of waste polyethylene terephthalate for upcycling and life-cycle assessment study. Nat. Commun..

[4] Myren T.H.T., Stinson T.A., Mast Z.J., Huntzinger C.G., Luca O.R. (2020). Chemical and electrochemical recycling of end-use poly(ethylene terephthalate) (PET) plastics in batch, microwave and electrochemical reactors. Molecules.

[5] Babaei M., Jalilian M., Shahbaz K. (2024). Chemical recycling of polyethylene terephthalate: A mini-review. J. Environ..

[6] Ren T.X., Zhan H.H., Xu H.Z., Chen L.F., Shen W., Xu Y.D., Zhao D.F., Shao Y.Y., Wang Y.T. (2024). Recycling and high-value utilization of polyethylene terephthalate wastes: A review. Environ. Res..

[7] Cusano I., Campagnolo L., Aurilia M., Costanzo S., Grizzuti N. (2023). Rheology of recycled PET. Materials.

[8] Zare Y. (2013). Recent progress on preparation and properties of nanocomposites from recycled polymers: A review. Waste. Manag..

[9] Lendvai L., Singh T., Ronkay F. (2024). Thermal, thermomechanical and structural properties of recycled polyethylene terephthalate (rPET)/waste marble dust composites. Heliyon.

[10] Wang D.R., Luo F.L., Shen Z.Y., Wu X.J., Qi Y.P. (2017). A study on the crystallization behavior and mechanical properties of poly(ethylene terephthalate) induced by chemical degradation nucleation. RSC Adv..

[11] Ravindra R.C., Katrin N.V., Florian R. (2018). Recycled poly(ethylene terephthalate)/clay nanocomposites: Rheology, thermal and mechanical properties. J. Polym. Environ..

[12] Hu W.B. (2018). The physics of polymer chain-folding. Phys. Rep..

[13] Lauritzen J.I.J., Hoffman J.D. (1960). Theory of Formation of Polymer Crystals with folded chains in dilute solution. J. Res. Natl. Bur. Stand. A Phys. Chem..

[14] Zhu Y.L., Liang C.S., Bo Y., Xu S.A. (2015). Non-isothermal crystallization behavior of compatibilized polypropylene/recycled polyethylene terephthalate blends. J. Therm. Anal. Calorim..

[15] Da Costa V., Le Moigne J., Oswald L., Pham T.A., Thierry A. (1998). Thin film orientation by epitaxy of carbazolyl polydiacetylenes:  guest−Host interaction on a crystal surface. Macromolecules.

[16] Liu Q., Zhang X.X., Jia D.Z., Yin J., Lei J., Xu L., Lin H., Zhong G.J., Li Z.M. (2023). In situ nanofibrillation of polypropylene/polyethylene/poly(ethylene terephthalate) ternary system: A strategy of upgrade recycling. Polymer.

[17] Sun Y.J., Li H.H., Huang Y., Chen E.Q., Gan Z.H., Yan S.K. (2006). Epitaxial crystallization of poly(butylene adipate) on highly oriented isotactic polypropylene thin film. Polymer.

[18] Yan S., Petermann J., Yang D. (1996). Epitaxial crystallization in the sPP/nylon-12 semicrystalline polymer system. Polymer.

[19] Wang B., Lin F.H., Li X.Y., Ji X.R., Liu S.X., Han X.J., Yuan Z.Q., Luo J. (2019). Transcrystallization of isotactic polypropylene/bacterial cellulose hamburger composite. Polymer.

[20] Wang B.J., Zhang Y.J., Zhang J.Q., Li H.Y., Chen P., Wang Z.B., Gu Q. (2013). Noncovalent method for improving the interaction between reduced graphene oxide and poly(ε-caprolactone). Ind. Eng. Chem. Res..

[21] Zhang X.X., Li Y.K., Sun Z.Y. (2018). Acceleration of crystal transformation from crystal form II to form I in polybutene-1 induced by nanoparticles. Polymer.

[22] Ouchiar S., Stoclet G., Cabaret C., Gloaguen V. (2016). Influence of the filler nature on the crystalline structure of polylactide-based nanocomposites: New insights into the nucleating effect. Macromolecules.

[23] Fujii L.C., Shiino M.Y. (2023). Development of polyurethane/polyethylene terephthalate/fiber glass polymeric composite from internal auto parts waste. Prog. Rubber Plast. Recycl. Technol..

[24] Ravindra R.C., Katrin N.V., Florian R. (2018). Recycled polyethylene terephthalate/carbon nanotube composites with improved processability and performance. J. Mater. Sci-Mater. M..

[25] Liang R., Chen Y.C., Zhang C.Q., Yin J., Liu X.L., Wang L.K., Kong R., Feng X., Yang J.J. (2017). Crystallization behavior of biodegradable poly(ethylene adipate) modulated by a benign nucleating agent: Zinc phenylphosphonate. Chin. J. Polym. Sci..

[26] Horrocks M., Kerscher C., Dotson D. (2008). A Novel Nucleating Agent for Polyethylene.

[27] Li Y.P., Guo Z.X., Xue M.L., Yan S.K. (2019). Epitaxial recrystallization of IPBu in Form II on an oriented IPS film initially induced by oriented form I IPBu. Macromolecules.

[28] Krivoshein P.K., Volkov D.S., Rogova O.B., Proskurnin M.A. (2020). FTIR photoacoustic spectroscopy for identification and assessment of soil components: Chernozems and their size fractions. Photoacoustics.

[29] Tkachenko Y., Niedzielski P. (2022). FTIR as a method for qualitative assessment of solid samples in geochemical research: A review. Molecules.

[30] Shen Z.Y., Luo F.L., Xing Q., Si P.F., Lei X.M., Ji L.J., Ding S.F., Wang K.Z. (2016). Effect of an aryl amide derivative on the crystallization behaviour and impact toughness of poly(ethylene terephthalate). CrystEngComm.

[31] Daubeny R.D.P., Bunn C.W., Brown C.J. (1954). The crystal structure of polyethylene terephthalate. Proc. R. Soc. Lond..

[32] Candal M.V., Safari M., Fernández M., Otaegi I., Múgica A., Zubitur M., Gerrica-Echevarria G., Sebastián V., Irusta S., Loaeza D. (2021). Structure and properties of reactively extruded opaque post-consumer recycled PET. Polymers.

[33] Avrami M. (1939). Kinetics of phase change. I general theory. J. Chem. Phys..

[34] Zhang X., Li L., Xie H., Liang Z.L., Su J.Y., Liu G.Q., Li B. (2013). Comparative analysis of thermal behavior, Isothermal crystallization kinetics and polymorphism of palm oil fractions. Molecules.

[35] Yang H.W., Du J.H. (2024). Crystallinity, rheology, and mechanical properties of low-/high-molecular-weight PLA blended systems. Molecules.

[36] Fillon B., Lotz B., Thierry A., Wittmann J.C. (1993). Self-nucleation and enhanced nucleation of polymers. Definition of a convenient calorimetric “efficiency scale” and evaluation of nucleating additives in isotactic polypropylene (α phase). J. Polym. Sci. Part B Polym. Phys..

[37] Saeed H.A.M., Eltahir Y.A., Xia Y., Wang Y. (2014). Non-isothermal crystallization kinetics and nucleation activity of hyperbranched polyester (HBPET) in recycled PET. Polym. Bull.

[38] Zhang R.C., Lu A., Xu Y., Min M., Xia J.Q., Zhou J.H., Huang Y.G., Li Z.M. (2009). Equilibrium melting temperature and spherulitic growth rate of poly(phenylene sulfide). Eur. Polym. J..

[39] Strobl G. (2000). From the melt via mesomorphic and granular crystalline layers to lamellar crystallites: A major route followed in polymer crystallization?. Eur. Phys. J. E.

[40] Li X.Y., Ding J.J., Chen P.J., Zheng K., Zhang X., Tian X.Y. (2021). Detection and characterization of folded-chain clusters in the structured melt of isotactic polypropylene. IUCrJ.

[41] Wang B., Nie K., Xue X.R., Lin F.H., Li X.Y., Xue Y.B., Luo J. (2018). Preparation of maleic anhydride grafted polybutene and its application in isotactic polybutene-1/microcrystalline cellulose composites. Polymers.

[42] Baldrian J., Steinhart M., Vlček P., Horký M., Laggner P., Amenitsch H., Bernstorff S. (2002). Time-resolved SAXS/WAXS study of phase behavior and crystallization in polymer blends. J. Macromol. Sci. B..

[43] Nonato R.C., Bonse B.C. (2016). A study of PP/PET composites: Factorial design, mechanical and thermal properties. Polym. Test..

